# Climate Change-Induced Decline in Succulent *Euphorbia* in Namibia’s Arid Regions

**DOI:** 10.3390/plants14020190

**Published:** 2025-01-11

**Authors:** J. J. Marion Meyer, Marie M. Potgieter, Nicole L. Meyer, Anika C. Meyer

**Affiliations:** Department of Plant and Soil Sciences, University of Pretoria, Hatfield, Pretoria P.O. Box X20, South Africanmeyer345@gmail.com (N.L.M.); anika.meyer345@gmail.com (A.C.M.)

**Keywords:** climate change, euphorbia, fairy circle, temperature, Namibia

## Abstract

The global rise in temperatures due to climate change has made it difficult even for specialised desert-adapted plant species to survive on sandy desert soils. Two of Namibia’s iconic desert-adapted plant species, *Welwitschia mirabilis* and the quiver tree *Aloidendron dichotomum*, have recently been shown to be under threat because of climate change. In the current study, three ecologically important Namibian *Euphorbia* milk bushes were evaluated for their climate change response. By comparing good-quality aerial photographs from the 1960s and recent 2020s high-resolution satellite images, it was determined by QGIS remote sensing techniques that very high percentages of the large succulents *E. damarana*, *E. gummifera*, and *E. gregaria* have died during the last 50 years in arid areas of Namibia. Areas like Brandberg (northern Namibia), Klein Karas (south-east), and Garub (south-west), with a high sandy-textured ground cover, have seen the loss of around 90% of *E. damarana* and *E. gregaria* and about 61% of *E. gummifera* in this period. This is alarming, as it could threaten the survival of several animal species adapted to feed on them, especially during droughts. This study focused on large succulent euphorbias, distinguishable in satellite images and historical photographs. It was observed that many other plant species are also severely stressed in arid sandy areas. The obtained results were ground-truthed and species identification was confirmed by the chemical analysis of remaining dead twigs using GC-MS and metabolomics. The ERA5 satellite’s 2 m above-ground temperature data show a 2 °C rise in annual average noon temperatures since 1950 at the three locations analysed. Annual daily temperatures increased by 1.3 °C since 1950, exceeding the global average rise of about 1.0 °C since 1900. This suggests that euphorbias and other plants on low-water-capacity sandy soils in Namibia face greater climate change pressure than plants globally.

## 1. Introduction

Over ninety percent of Namibia’s land surface is characterized as semi-arid, arid, or hyper-arid, making it the driest country in Africa south of the equator. Consequently, Namibia experiences limited precipitation, and droughts frequently occur across much of the country [1]. Namibia is situated amidst two deserts, with the Namib Desert to its west and the Kalahari Desert to its east. It is bordered by several rivers that flow year-round: the Zambezi and Okavango Rivers to the north-east, the Kunene River to the north-west, and the Orange River to the south. The remaining rivers within Namibia are ephemeral, flowing only briefly during some of the rainy seasons [2].

**Figure 1:**
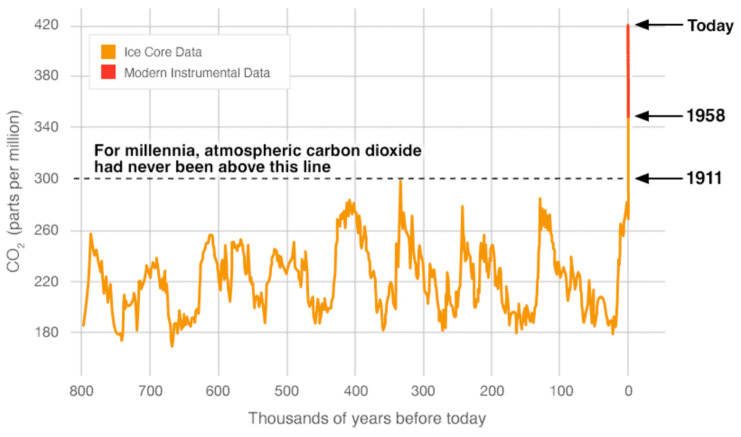
CO_2_ levels during Earth’s last three glacial cycles, as captured by air trapped in ice sheets and glaciers [3].

Long-term shifts in weather patterns have been unnaturally accelerated by unsustainable human activities since the industrial revolution, resulting in larger quantities of carbon dioxide (CO_2_), methane (CH_4_), nitrous oxide, and other heat-absorbing gasses (Figure 1, [3]). Increasing levels of these greenhouse gasses trap more heat in the Earth’s atmosphere, causing global temperatures to rise and leading to warmer climates. The global average temperature in 2023 was approximately 1.36 °C higher than the preindustrial average of 1850–1900 [3], surpassing the previous warmest year of 2016, which had a temperature increase of 1.29 °C. In 2022, atmospheric greenhouse gas levels reached record highs: CO_2_ averaged 418 parts per million (ppm), CH_4_ 1923 parts per billion (ppb), and N_2_O stood at 336 ppb, respectively representing 150%, 266%, and 124% of pre-industrial (1750) levels. Although CO_2_ grew at 2.2 ppm, slightly below the 10-year average of 2.46 ppm/year, this rate is commonly lower in La Niña years like 2022, compared to El Niño years like 2016.

It was stated by Bombi et al. [4] that deserts are predicted to be highly responsive to global climate change. These authors investigated the spatial and demographic responses of *Welwitschia mirabilis*, a keystone endemic plant of the Namib Desert, to potential displacement and reduced suitable habitats under future climate scenarios. They found that welwitschia’s historically suitable thermal niche in northern Namibia will disappear almost entirely within 30 years. Declining climatic suitability in Namibian study sites strongly correlated with negative population indicators, including poor plant health, reduced recruitment, and higher adult mortality. They concluded by recommending the establishment of a large-scale programme to identify sentinel species for climate change. This would enable the better detection, monitoring, and assessment of climate change impacts on biodiversity, enhancing long-term ecosystem-level conservation.

The effect of climate change on the vegetation of Namibia was also investigated by Foden et al. [5]. These authors used *Aloidendron dichotomum* (=*Aloe dichotoma*, quiver trees) as an indicator species to demonstrate how climate change is causing a population decline in arid regions of southern Africa. They concluded that desert ecosystems are likely to become increasingly hostile to endemic plants and animals, and thus more species-poor with intensifying warming [5].

**Figure 2:**
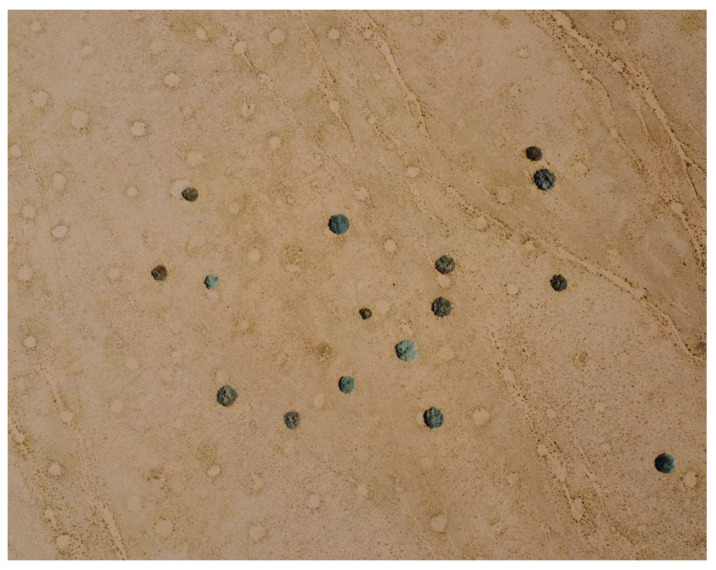
Nearly all of these last-remaining *Euphorbia damarana* plants growing on sandy soil in Brandberg were dead in 2023. Many fairy circles (barren circular patches devoid of vegetation) can be seen between the euphorbias in this aerial photograph. It has been hypothesised by Meyer et al. [6] that fairy circles are caused by dead *E. damarana* and other large succulent *Euphorbia* species.

It was briefly mentioned by Meyer et al. [6] that many large succulents like *Euphorbia damarana*, *E. gummifera*, and *E. gregaria* (milk bushes) were observed to be dead, with many more in the process of dying (Figure 2), on the plains of sandy soil in Namibia’s extremely arid regions. Thousands of fairy circles are visible when there is sufficient grass cover in sandy areas where euphorbias have died in large numbers. Although their milky latex is poisonous, several animal species have adapted to feed on them, including oryx, kudu, springbok, and black rhinoceros [7,8]. Since these milk bushes are often the only vegetation available to many browsing species during droughts, the survival of these plants is critical.

In the current study, we provide quantitative data on the substantial mortality of *E. damarana*, *E. gummifera*, and *E. gregaria* in sandy areas of the northern (Brandberg), south-eastern (Garub), and south-western (Klein Karas) arid parts of Namibia, respectively. Additionally, we present ERA5 temperature data that reveal a significant increase in temperature in these arid regions of Namibia since 1950.

## 2. Materials and Methods

### 2.1. Plant Collection and Identification

**Figure 3:**
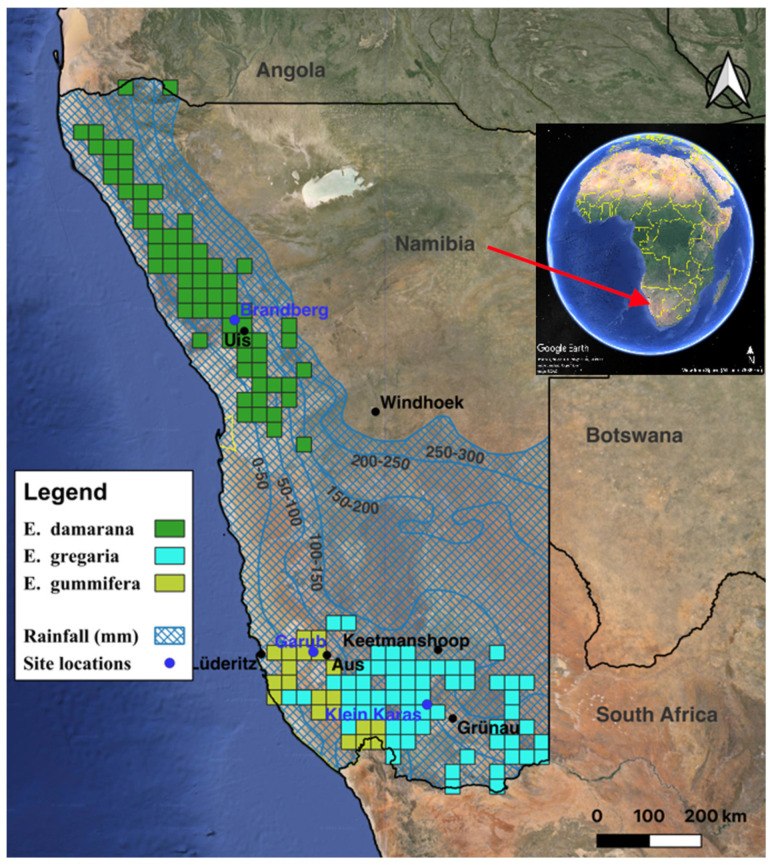
Distribution of *Euphorbia damarana*, *E. gummifera*, and *E. gregaria*; the site locations; and rainfall isohyets of the arid regions in Namibia [9] (data also added from NBRI and SANBI).

*Euphorbia damarana* L. C. Leach, *E. gummifera* Boiss., and *E. gregaria* Marloth were collected in Namibia from Brandberg, Garub, and Klein Karas, respectively. The specimens were identified by Ms Magda Nel at the H.G.W.J. Schweikerdt Herbarium of the University of Pretoria, South Africa (*E. damarana*, voucher PRU 122228; *E. gummifera*, voucher PRU 124383; *E. gregaria* was identified by comparison to several previously collected voucher specimens, including voucher PRU 124852). A distribution map of these three species, the study sites’ locations, and the mean annual rainfall of the arid regions where they are found, are shown in Figure 3. The data for the distribution of *E. damarana*, *E. gummifera*, and *E. gregaria* are from the Tree Atlas Project of Namibia [9], the National Botanical Research Institute (NBRI) of Namibia, and the South African National Biodiversity Institute (SANBI), as well as some of our own observations. The data were collected by grid cells (15′ × 15′ or quarter-degree square, approx. 27 × 27 km).

### 2.2. Euphorbia Population Decline

#### 2.2.1. Historical Photograph Analysis

The assessment of the decline in the *Euphorbia* population was conducted by georeferencing (QGIS 3.38) and comparing good-quality 1:36,000-scale historical aerial photographs from 1967 to 1969 (Ministry of Agriculture, Water and Land Reform, Namibia) to recent satellite images (DigitalGlobe-Maxar; four bands: near-infrared, red, green, and blue; 1.5 m resolution; Google Earth Pro’s DigitalGlobe-Maxar and Airbus 2010 and 2023 images). Since the aerial parts of the *Euphorbia* species consist nearly exclusively of evergreen stems and very few extremely small leaves, the comparison between historical photographs and satellite images was not influenced by resolution differences or seasonal effects. Analyses were performed at the following three randomly chosen sites, which are representative of the three large succulent species of Namibia and stretch from the northern to the most southern areas of the country (Figure 3):

- Brandberg, north-western Namibia, *E. damarana*, north-west corner of site Brandberg1 (−21.0532306 N, 14.7455993 E) and site Brandberg2 (−21.0530105 N, 14.7535729 E).
- Garub, south-western Namibia, *E. gummifera*, north-west corner of site Garub1 (−26.628371 N, 16.054868 E) and site Garub2 (−26.6009390 N, 16.0138877 E).
- Klein Karas, south-eastern Namibia, *E. gregaria* north-west corner of site Klein Karas1 (−27.493063 N, 17.944561 E) and site Klein Karas2 (−27.496656 N, 17.943603 E).

The landcover of all these sites are predominantly desert dune sand [10] and 2.5–5.0 hectares in size, depending on the number of euphorbias present at the location. Ground-truthing covering all of the remote sensing analysis sites was undertaken from 2022 to 2024.

#### 2.2.2. Determination of the Number of Seedlings and Dead Plants Not Detectable Through Remote Sensing

The number of *E. damarana* seedlings and dead plants in Brandberg, including plants with minimal remaining dead plant parts not visible on aerial photographs and satellite images, was determined. All plants with only a few dead remains that were not visually identifiable to species level were collected in plastic honey jars and analysed by Gas Chromatography–Mass Spectroscopy (GC-MS) to look for euphorbia biomarker compounds. This part of the study was conducted across 5-hectare sites of desert dune sand (DesertDS; NW corner −21.0536574 N, 14.7536136 E), grassland (Grassl; −20.9518574 N, 14.6314255 E) and rocky outcrops on hills (RockyO; −21.1341229 N, 14.8526790 E) to determine if mortality varied across these landcover types [10].

The extraction of compounds from the plants’ dead remains, GC-MS, and metabolomic analyses were conducted as described in Degashu et al. [7]. GC-MS data were processed and analysed using MestReNova 14.2 (Mestrelab Research, Santiago de Compostela, Spain) and the database of the National Institute of Standards and Technology vers. 14 (NIST) was used for compound identification. The biomarker euphol was previously detected in *E. damarana* [7], and its presence or absence was determined in the remains of the collected dead plant samples. For metabolomic analysis [8], each spectrum was referenced individually against the solvent peak methanol. The data were binned at 0.04 ppm intervals, and principal component analysis (PCA) and orthogonal partial least squares discriminant analysis (OPLS-DA) models were created using SIMCA 1.4 (Sartorius). Both models were Pareto-scaled.

### 2.3. Normalized Difference Vegetation Index (NDVI) Analysis

NDVI analysis was conducted to determine if healthy *E. damarana* plants in the Brandberg region deteriorated between 2012 and 2021. Since the NDVI is dependent on the concentration of chlorophyll in plants, the index value is directly related to the photosynthetic activity of plants [11]. Band and layer rendering properties were adjusted on two 1.5 m resolution multiband satellite images (DigitalGlobe-Maxar) from 2012 and 2021 to create false-colour composites to distinguish between living and dead *E. damarana* plants. These were ground-truthed in 2022. Only *E. damarana* plants alive in 2012 and larger than 4 m in diameter were individually delineated as polygon shape files, and the average NDVI calculated for 2012 and 2021. The polygon shapefile raster data were extracted from the satellite image to a mask layer and a raster calculator was used to determine the NDVI values using band 3 (red light) and band 4 (near-infrared light). The analysis was conducted for the following three 25-hectare sites in the sandy areas [10] of Brandberg: NW corners of Grassland1 −20.9497428 N, 14.6314007 E; Grassland2 −20.9375687 N, 14.6307963 E; and DesertDune −21.1284304 N, 14.7930459 E. Student’s *t*-test, degrees of freedom, and *p*-values were determined.

### 2.4. Temperature Data

The daily 2 m above-ground temperatures at 12:00 noon and the daily average temperatures from 1950 to 2023 were obtained from the ERA5 reanalysis hourly data on single levels dataset [12].

## 3. Results

Since the three large Namibian *Euphorbia* species, *E. damarana*, *E. gummifera*, and *E. gregaria*, were previously observed to be dying in large numbers and could be monitored by remote sensing using historical aerial photographs and satellite images, the focus was on them in this study. It was, however, also noticed that several other plant species were dying in unusually large numbers, but they are not reported on in this study.

Samples of the dying *Euphorbia* species were collected and visually inspected using stereo and light microscopy for the presence of pathogen damage at the Forestry and Agricultural Biotechnology Institute, University of Pretoria, but no link to pathogens that might have caused their deteriorating health was observed.

### 3.1. Historical Photograph Analysis

**Figure 4:**
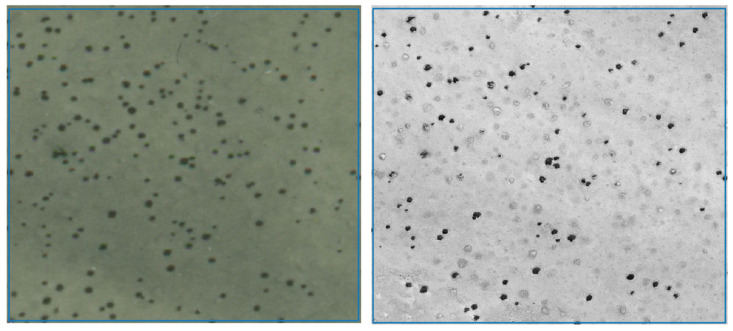
Site Garub2: Historical 1967 aerial photograph (**left**) and recent satellite image (**right**) showing that 57.4% of *E. gummifera* plants have died since 1967.

**Figure 5:**
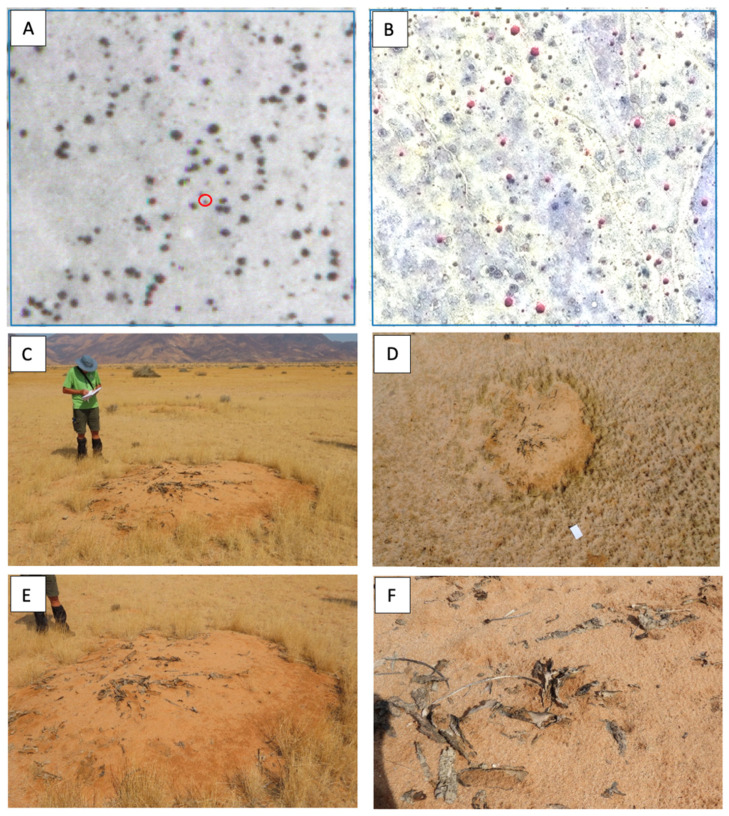
Site Brandberg1: Historical 1969 aerial photograph (**A**) and recent satellite image (false-colour band 342 (**B**)) showing that 87.2% of *E. damarana* plants have died since 1969. Typical circular barren patch (fairy circle) of a dead *E. damarana* plant ((**C**), marked with red circle in (**A**)) with its remains still visible. The same circular barren patch as seen on a drone photograph (**D**), the same barren patch enlarged (**E**), and the *E. damarana* remains enlarged (**F**). The remains were analysed by GC-MS and confirmed to be from an *E. damarana* plant.

**Figure 6:**
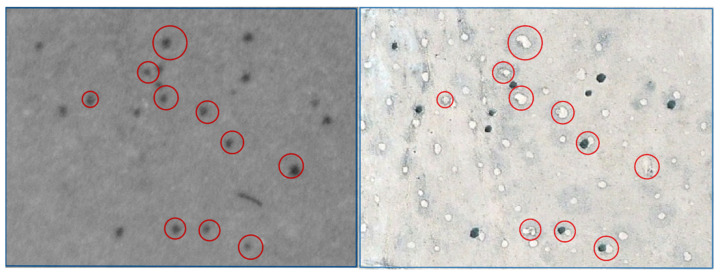
An enlarged section of a Brandberg site with 20 *E. damarana* plants in the historical 1969 photograph (**left**). Recent satellite image (**right**) showing that 10 of the 20 *E. damarana* plants (red circles) have died since 1969 and formed bare patches with no grass cover, forming new fairy circles.

**Figure 7:**
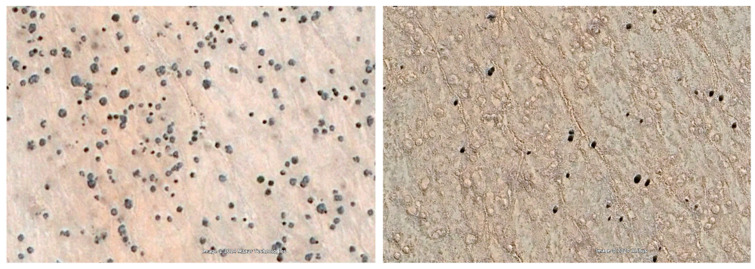
The Klein Karas2 site showing an abundance of *E. gregaria* plants in a Google Earth satellite image from 2010 (**left**). Many of these plants were probably already dead (black vs. intense blue-green colour) in 2010. About 89.9% of these euphorbias formed barren patches (new fairy circles), as can be seen in the 2023 satellite image (**right**).

The comparison of historical aerial photographs from 1967 to 1969 with recent satellite images of 1.5 m resolution showed remarkably high mortality rates among *E. damarana* in Brandberg (91.3%, average of two sites), *E. gummifera* in Garub (61.4%), and *E. gregaria* in Klein Karas (88.1%) over the past 50 years (Table 1, Figure 4, Figure 5, Figure 6 and Figure 7). A further study would be required to investigate why the mortality rate of *E. gummifera* was notably less, as determined in Garub, than that of the other two species. The answer may lie in a complex interplay of various environmental factors (including geology) and species resilience. The significant temperature increase of approximately 2 °C at all three sites (see Section 3.4 below) was likely a major factor contributing to the high mortality rates of all three milk bush species.

### 3.2. Presence of Seedlings and the Mortality of Euphorbias Not Detectable Through Remote Sensing

Remarkably, only two seedlings (diameter smaller than 1.0 m) were recorded for *E. damarana* across the two Brandberg sites, and no *E. gummifera* seedlings were found in either site at Garub. In Klein Karas, only five seedlings were found across the two sites. In a study by Lewandrowski et al. [13], the authors demonstrated that the transition from seed to established seedling is highly vulnerable to increases in temperature under water-limited conditions, representing a critical filter for plant recruitment in arid zones. They showed that an increase in temperature to 35–40 °C under water-limited conditions resulted in a 95–100% failure in seed recruitment, regardless of seed dormancy status.

**Figure 8:**
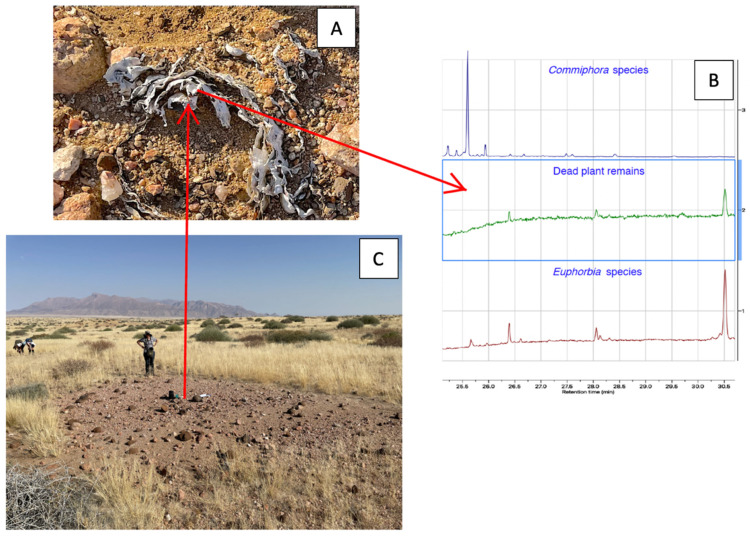
To identify the plant species that previously grew in newly developing fairy circles (**C**), dead plant remains (**A**) were analysed using GC-MS (**B**). The resulting GC-MS chromatogram and mass spectra of the dead plant remains (middle chromatogram of (**B**)) showed the presence of the toxic triterpenoid euphol at 30.5 min., a characteristic *Euphorbia* compound [7]. It was also detected in all other dead plant samples collected from circular barren patches (newly forming fairy circles). *E. damarana* plants can be seen in the background of photograph (**C**) taken in Brandberg, Namibia. The chromatogram (top of (**B**)) of one of the other plant species found in the area, a *Commiphora* species, was quite different and did not contain euphol.

**Figure 9:**
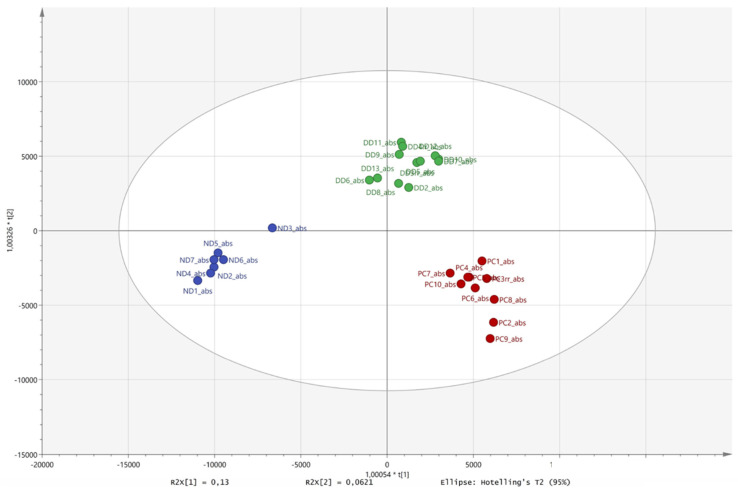
The OPLS-DA grouping of the GC-MS metabolomic comparison of the dead remains from circular barren patches (green), *E. damarana* plants (red), and remains collected from areas which were not inhibiting grass growth (blue). The model’s statistical parameters showed that the data fitted the plot very well (R2X = 0.93; R2Y = 0.94 and Q2 = 0.68).

The dead euphorbias in Brandberg with only a few remaining dried parts were not visible on aerial photographs or satellite images, and were therefore quantified by fieldwork. The collected dead remains from circular barren patches (fairy circles) could be conclusively identified as those of *E. damarana* by their tubular stem shapes and chemical analyses (GC-MS and metabolomics, Figure 8 and Figure 9). The GC-MS chromatogram and the mass spectra [7] of the dead plant remains collected from newly formed fairy circles conclusively showed the presence of the toxic triterpenoid euphol, a typical *Euphorbia* compound, at a retention time of 30.5 min (Figure 8). It was also detected in all the other dead plant samples collected from newly formed fairy circles.

A few grass tufts were sometimes present inside some of the newly formed fairy circles, but only if nebkhas were still present inside them. These nebkhas are formed by euphorbias’ collection of aeolian sand particles over their lifetime [6]. No grasses were observed when the surface of the fairy circle was already flat. This phenomenon is likely due to the combined effect of the milky latex’s toxicity and hydrophobicity in both the roots and the aerial parts of the plant, which are necessary to achieve the full allelopathic effect [6].

The metabolomic GC-MS-based OPLS-DA analysis (Figure 9) of the (1) dead remains collected from circular barren patches, (2) *E. damarana* plants, and (3) dead plant remains collected from areas which were not inhibiting grass growth, only showed a close similarity between the chemistry of the (1) dead remains from inside new fairy circles and (2) *E. damarana* plants. The model’s statistical parameters of analysis showed that the data fitted the plot very well (R2X = 0.93; R2Y = 0.94 and Q2 = 0.68).

**Figure 10:**
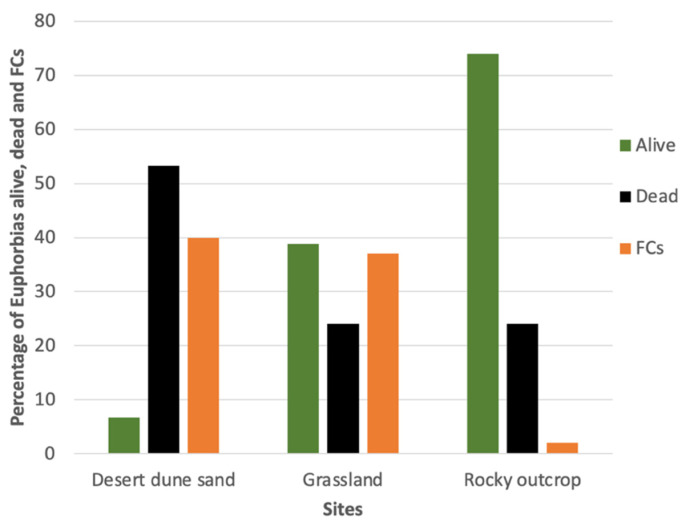
The percentage of alive and dead *E. damarana* plants, as well as fairy circles (FCs) found during onsite fieldwork analysis in three sites of different landcover classes in the Brandberg area [10].

The percentage of living *E. damarana* plants was found to be ten times higher in the rocky outcrop landcover site [10] than in the desert dune sand landcover sites (Figure 10) during the onsite fieldwork analysis. This is most likely due to the higher evaporation and drainage rates and much lower water-holding capacity of sandy soil compared to rocky outcrops, which contain a much higher percentage of clay soil. These stress effects of sandy soils are probably much enhanced by rising temperatures. The percentage of fairy circles was about 20 times higher in the two sandy sites than in the rocky outcrop site, confirming previous reports of fairy circles occurring mainly on sandy soil [6,14].

### 3.3. NDVI Analysis

**Figure 11:**
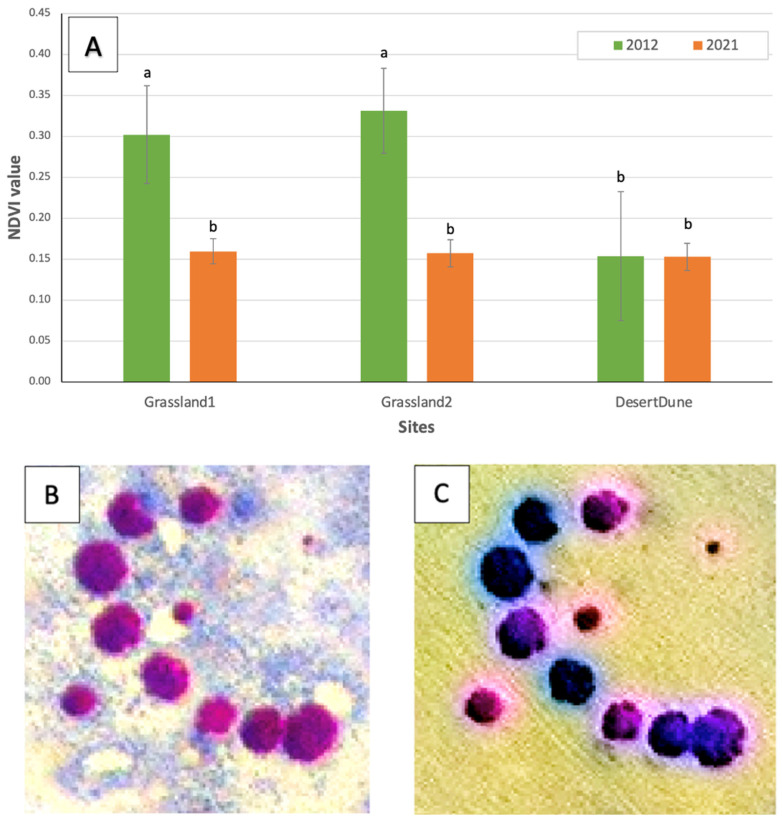
NDVI values of *E. damarana* plants in the two grassland landcover cites (Grassland1 and Grassland2) and the desert dune sand site (DesertDune). Different letters indicate statistically significant differences (*p* < 0.05) (**A**). The health of the euphorbias declined by about 50% from 2012 to 2021 in the two grassland sites, whereas the *Euphorbia damarana* were already mostly dead in 2012 in the desert dune sand site. An example of the decline in *E. damarana* health is shown in false-colour satellite images: 2012’s NDVI = 0.34 (**B**); 2021’s NDVI = 0.17 (**C**). Darker colours indicate less functional chlorophyll.

The NDVI values of the *E. damarana* plants in Brandberg’s two grassland landcover sites (Grassland1 and Grassland2) both decreased by about 50% from 2012 to 2021 (Figure 11A). Since the NDVI is directly related to chlorophyll content, the general health of the plants in the two grassland sites declined by about half. In the desert dune sand landcover site (DesertDune), the NDVI value of 2012 was already very low (0.15), indicating that nearly all the plants were already in an unhealthy condition in 2012.

### 3.4. ERA5 Satellite Temperature Data

**Figure 12:**
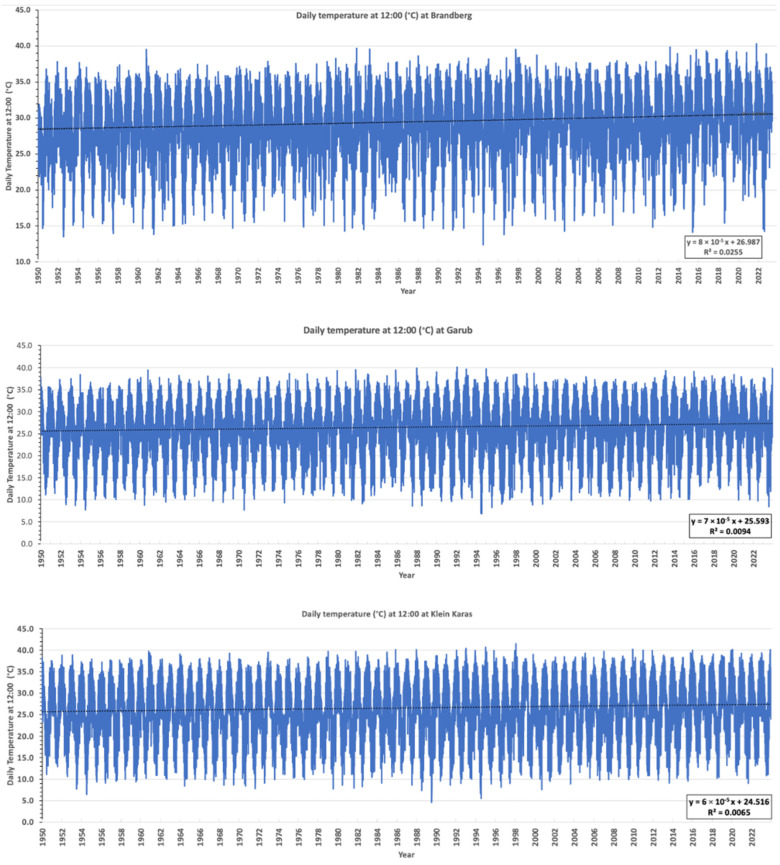
The daily temperature at 12:00 increased by about 2 °C since 1950 in Brandberg (**top**), Garub (**centre**), and Klein Karas (**bottom**). Temperatures were obtained from ERA5 reanalysis hourly data on single levels dataset [12].

**Figure 13:**
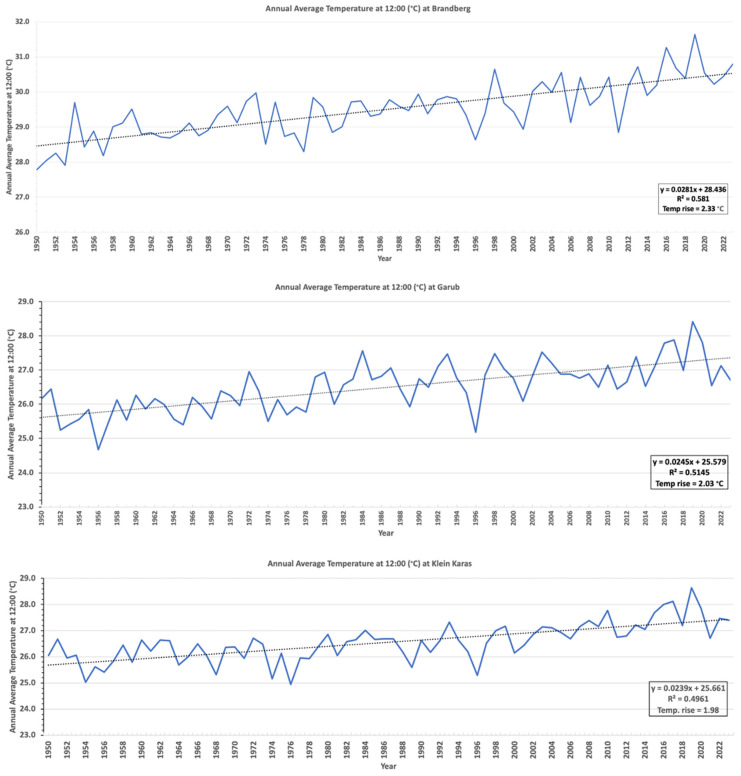
The average annual temperature at 12:00 in Brandberg (**top**), Garub (**centre**), and Klein Karas (**bottom**) has risen by about 2 °C since 1950 (ERA5 reanalysis hourly data on single levels dataset, [12]).

The daily temperature (at 2 m) at 12:00 noon from 1950 to 2023 at the study sites in Brandberg, Garub, and Klein Karas (Figure 12) was extracted from the ERA5 reanalysis hourly data on single levels dataset [12]. The average annual temperature at 12:00 at all three locations has risen by about 2 °C since 1950 (Figure 12). The average annual daily temperature increased by about 1.3 °C in these three areas (Figure 13), which is significantly higher than the global 1.0 °C increase since 1900 [15,16,17]. This indicates that the euphorbias and other species growing on the sandy soils with very low water retention in the arid areas of Namibia are probably under considerably more pressure than plants globally because of climate change.

## 4. Discussion

This study’s onsite analysis and comparison of historical photographs and recent satellite images of the sandy and arid regions of Namibia have shown that the numbers of *E. damarana*, *E. gummifera*, and *E. gregaria* have declined sharply since 1950. Very few seedlings are presently found in these areas. Although these milk bushes are toxic to humans and livestock [7], various indigenous animals have remarkably adapted to consume their succulent branches and fruit. Springbok, oryx, kudu, Hartmann’s mountain zebra, and black rhino browse on these plants, while unconfirmed reports from locals also suggest that klipspringer, steenbok, baboons, and porcupines consume their stems and/or fruit [8]. During droughts, these toxic succulents become a crucial source of food and moisture for herbivores. The Namibian oryx, for example, derives more than 25 percent of its diet from the Damara milk bush during droughts [18]. Since the average daily temperature at noon since 1950 has increased by about 2 °C, and the average annual temperature by about 1.3 °C, considerably higher than the global average, these milk bushes seem to be exposed to harsh environmental conditions, especially given their substrate is sandy soil. It is likely that the declining numbers of these milk bushes will have a detrimental effect on several animal species, which could worsen if climate change continues at its current pace.

In addition to the significant temperature increase and the striking decline in Namibia’s iconic desert-adapted *Euphorbia* succulents reported in this study, climate change has also affected rainfall patterns in the arid western regions of southern Africa. Attwood et al. [19] established that the frequency of strong heat low days is an important feature in determining increasing aridity trends over southern Africa. They analysed ERA5 ECMWF reanalysis data and found a rapidly increasing frequency of strong heat low days, with a 175% increase between 1960–1989 and 1990–2019. A significant increase of 459% was found during the early summer months of September/October, aligning with forecasts of a delayed start to the rainy season. More strong heat lows were detected in the most recent 5 years of analysis (2014–2019) than in the 30-year period from 1960 to 1989. A continuation of this trend could result in a shortening of the rainy season over Africa [19].

None of the common anthropogenic factors leading to plant population decline, such as invasive species, urbanisation, expanding agriculture, or overgrazing, were observed to impact the decline of the three *Euphorbia* populations in Namibia. Similarly, activities like mining, quarrying, illegal plant collection, or pollution do not seem to contribute to their decline.

While climate change is a serious current concern, it has also been a recurring event throughout prehistoric times. Scott [20] used middens of herbivorous rock hyrax (*Procavia capensis*) and dassie rat (*Petromus typicus*) preserved in rocky areas of the Kuiseb riverbed in the central part of the Namib Desert to explore the paleoenvironmental history of the area. Plant remains such as pollen grains become sealed and embedded in the dried urine of fossil middens. Pollen in old midden layers not only reflects vegetation cover, but also past climatic conditions [20,21,22,23].

**Figure 14:**
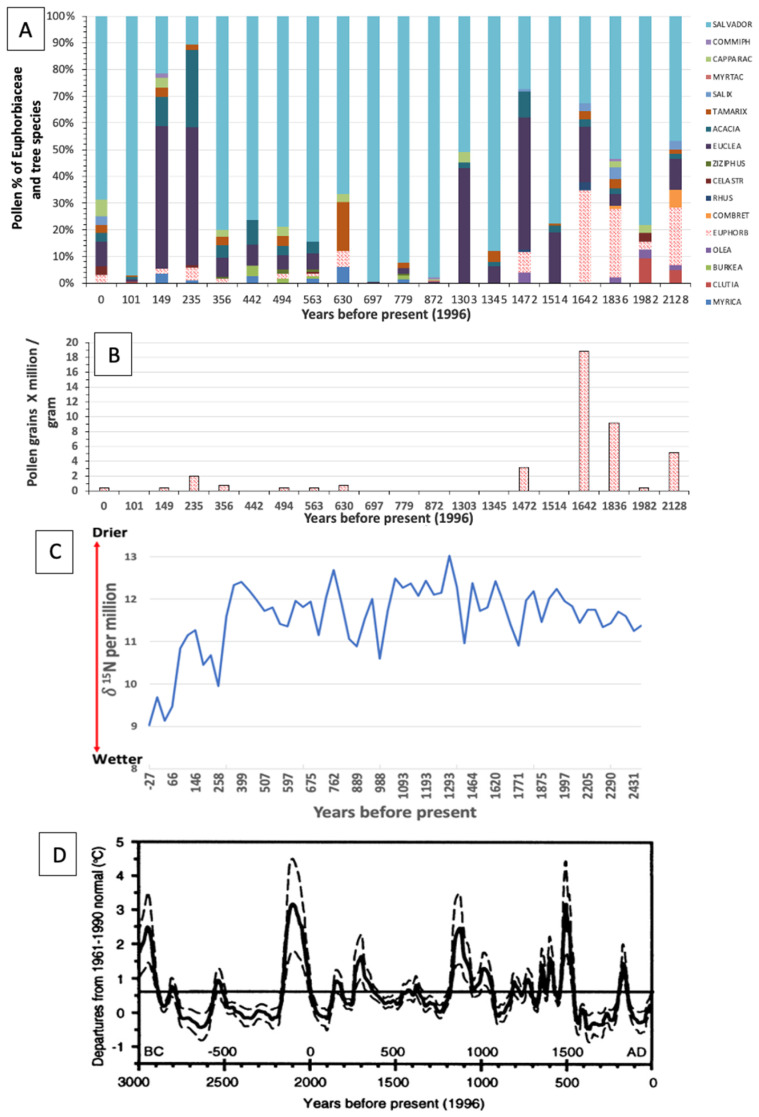
(**A**) Pollen records from rock hyrax and dassie rat middens in the Kuiseb Valley (Namib Sand Sea), showing a marked decline in *Euphorbia* pollen (red dotted) from about 1400 before present (BP) (adapted from Scott [20]). (**B**) Pollen record of Euphorbiaceae species extracted from (**A**). (**C**) The driest period during the last approx. 2500 years in Namibia (Spitzkoppe) is coinciding approximately with the big decline in *Euphorbia* pollen between 400 and 1350 BP (adapted from Chase et al. [25]), and (**D**) showing above average high temperatures from 500 to 1200 BP [24].

Scott [20] found that the pollen quantity of *Euphorbia* species declined sharply (Figure 14A,B) in rock hyrax and dassie rat dung from about 1400 years ago to the current time in the Kuiseb Valley bordering the Namib Sand Sea (raw data kindly made available by L. Scott). During part of this time (1400–400 before present (BP)), southern Africa experienced a prolonged period of above-average temperatures, along with intervals of extreme heat (Figure 14D) [24] and also the driest period during the last 2500 years (Figure 14C) [25]. These extremes, as well as the low water-holding capacity of sand, could have been major contributors to the decline in the populations of *Euphorbia* species in the Namib Desert. Scott [20] also attributed the pollen fluctuations observed over time in some species/families to climate change. The Euphorbiaceae pollen data (kindly made available by L. Scott) from fossilised middens near Pella [26], close to the Namibia–South Africa border, also show a marked decline at around 1400 BP. The large succulent milk bush *E. gregaria* is found in this area [6]. *Euphorbia* pollen was also identified in rock hyrax middens in Zizou on the eastern margin of the Namib Sand Sea [21]. It was stated that it is generally considered that rock hyrax middens preserve a predominantly local signal that reflects the foraging range of the animals and vegetation in the Namib Desert [21].

In the areas where Scott [20] and Chase et al. [21] collected midden samples for pollen analyses, thousands of fairy circles are currently found, but no large succulent *Euphorbia* species that could have caused them. It is, however, hypothesised [6] that fairy circles, including those in the northern and eastern borders of the Namib Sand Sea, are caused by populations of large succulent euphorbias, probably *E. gummifera* or *E. damarana*, that died because of climate change. The nearest current location of *E. damarana* to Scott’s collection site is about 50–100 km to the northeast, and the nearest *E. gummifera* plants are about 180 km south [27]. A reasonable question to ask is therefore whether the millions of fairy circles in Namibia can serve as visual markers (‘footprints’ or ‘tombstones’) of current or even historical climate change, which may have led to the widespread death of these species in sandy areas. In the current study, the high numbers of dying *Euphorbia* species found in areas where fairy circles occur is also ascribed to climate change. These milk bushes, and probably also other large species containing resin or latex, occurring in arid sandy areas of the world, will cause fairy circles after they die. These ‘footprints’ could potentially be used to estimate former population sizes and plant distributions and serve as possible indicators of climate change.

Although *Euphorbia* species have adapted to survive in environments marked by high temperatures, extended and recurrent droughts and escalating temperatures have now also caused them to be under threat of extinction in some areas of Morocco [28,29]. The vulnerability of *Euphorbia* ecosystems, which are uniquely adapted to hostile environments, is currently in urgent need of a strategic and timely conservation response [30]. In the face of climate change, *Euphorbia* species hold potential as an ideal choice for urban landscaping in arid regions due to their resilience and adaptability [31].

## 5. Conclusions

The ERA5 temperature data analysed in this study showed that temperatures have risen by an alarming 2 °C at noon in the arid regions of Namibia since 1950. This study also reports that three of the hardiest, desert-adapted CAM photosynthesising succulent *Euphorbia* species, *E. damarana*, *E. gregaria*, and *E. gummifera*, have experienced a significant decline in numbers in the sandy, nutrient-poor soils of these regions. The significant temperature increase, along with recent droughts, probably played a direct role in this decline. During the ground-truthing of dead *Euphorbia* species and onsite fieldwork, barren circular areas (fairy circles) were constantly observed where their remains were found. The dead plant remains found in these newly formed fairy circles could conclusively be identified as *Euphorbia* species from their morphology and GC-MS analyses. The results of this study demonstrated that, similar to *Welwitschia mirabilis* [4] and *Aloidendron dichotomum* [5], three other Namibian desert-adapted succulents, *E. damarana*, *E. gregaria*, and *E. gummifera*, are also under threat, especially in sandy areas, due to climate change.

## Figures and Tables

**Table 1:** Decline in *E. damarana* (Brandberg), *E. gummifera* (Garub), and *E. gregaria* (Klein Karas). The number of plants that died was determined by comparison of historical aerial photographs and satellite images. Since no historical aerial photographs were available for Klein Karas, comparisons were made between 2010 and 2023 satellite images available on Google Earth Pro.

Site Name	Historical Photo (Year)	Euphorbias Visible on Historical Photos	Euphorbias Visible on Recent Satellite Images	Euphorbias not Visible (Died) on Satellite Images	Percentage of Dead Euphorbias
Garub1	1967	185	64	121	65.4
Garub2	1967	176	92	101	57.4
Brandberg1	1969	133	17	116	87.2
Brandberg2	1969	149	7	142	95.3
Klein Karas1	2010	255	35	220	86.3
Klein Karas2	2010	287	29	258	89.9

## Data Availability

The original contributions presented in this study are included in the article. Further inquiries can be directed to the corresponding author.

## References

[1] Mapani B.S., Shikangalah R.N., Mwetulundila A.L. (2023). A review on water security and management under climate change conditions, Windhoek, Namibia. J. Afr. Earth Sci..

[2] Mendelsohn J., Jarvis A., Roberts C., Robertson T. (2002). Atlas of Namibia.

[3] (2024). NASA Climate Change. https://climate.nasa.gov/vital-signs/carbon-dioxide/?intent=121.

[4] Bombi P., Salvi D., Shuuya T., Vignoli L., Wassenaar T. (2021). Climate change effects on desert ecosystems: A case study on the keystone species of the Namib Desert *Welwitschia mirabilis*. PLoS ONE.

[5] Foden W., Midgley G.F., Hughes G.A., Bond W.J., Thuiller W., Hoffman M.T., Prince Kaleme P., Underhill L.G., Rebelo A., Lee H. (2007). A changing climate is eroding the geographical range of the Namib Desert tree Aloe through population declines and dispersal lags. Divers. Distrib..

[6] Meyer J.J.M., Schutte C.E., Hurter J.W., Galt N.S., Degashu P., Breetzke G., Baranenko D., Meyer N.L. (2020). The allelopathic, adhesive, hydrophobic and toxic latex of *Euphorbia* species is the cause of fairy circles investigated at several locations in Namibia. BMC Ecol..

[7] Degashu M.P., Meyer J.J.M., Alberts P.S.F., Meyer N.L., Blignaut M., Makhaba M., Hussein A.A. (2024). Isolation and identification of the primary toxin in the smoke of the Namibian milk bush, *Euphorbia damarana*. SA J. Bot..

[8] Meyer J.J.M., Meyer A.C., Meyer N.L. (2020). Sand circles in stony landscapes of Namibia are caused by large *Euphorbia* shrubs. SA J. Bot..

[9] Curtis B., Mannheimer C. (2005). Tree Atlas of Namibia.

[10] (2023). Digital Atlas of Namibia. https://www.unikoeln.de/sfb389/e/e1/download/atlas_namibia/index_e.htm.

[11] Hamlyn G.J., Robin A.V. (2010). Remote Sensing of Vegetation. Principles, Techniques, and Applications.

[12] (2024). Copernicus Climate Change Service. https://confluence.ecmwf.int/display/CKB/ERA5%3A+data+documentation.

[13] Lewandrowski W., Stevens J.C., Webber B.L., Dalziell E.L., Trudgen M.S., Bateman A.M., Todd E.E. (2021). Global change impacts on arid zone ecosystems: Seedling establishment processes are threatened by temperature and water stress. Ecol. Evol..

[14] Meyer J.J.M., Schutte C.S., Galt N., Hurter J.W., Meyer N.L. (2021). The fairy circles (circular barren patches) of the Namib Desert - What do we know about their cause 50 years after their first description?. S. Afr. J. Bot..

[15] (2024). NASA Earth Observatory. https://earthobservatory.nasa.gov/world-of-change/global-temperatures.

[16] (2024). NASA GISS. https://climate.nasa.gov/vital-signs/global-temperature/?intent=121.

[17] (2024). US EPA. https://www.epa.gov/climate-indicators/climate-change-indicators-us-and-global-temperature.

[18] Lehmann D., Kazgeba J., Mfune E., Gewers E., Cloete J., Brain J., Voigt C. (2013). Dietary Plasticity of Generalist and Specialist Ungulates in the Namibian Desert: A Stable Isotopes Approach. PLoS ONE.

[19] Attwood K., Washington R., Munday C. (2024). The southern African heat low: Structure, seasonal and diurnal variability, and climatological trends. J. Climatol..

[20] Scott L. (1996). Palynology of hyrax middens: 2000 years of palaeoenvironmental history in Namibia. Quat. Int..

[21] Chase B.M., Boom A., Carr A.S., Meadows M.E., Lim S. (2023). A ca. 39,000-year record of vegetation and climate change from the margin of the Namib Sand Sea. Quat. Res..

[22] Scott L., Bousman C.B. (1990). Palynological analysis of hyrax middens from southern Africa. Paleogeogr. Paleoclimatol. Paleoecol..

[23] Scott L., Cooremans B. (1992). Pollen and recent *Procavia* (hyrax), *Petromus* (dassie rat) and bind dung in South Africa. J. Biogeogr..

[24] Holmgren K., Tyson P.D., Moberga A., Svanered O. (2001). A preliminary 3000-year regional temperature reconstruction for South Africa. S. Afr. J. Sci..

[25] Chase B.M., Meadows M.E., Scott L., Thomas D.S.G., Marais E., Sealy J., Reimer P.J. (2009). A record of rapid Holocene climate change preserved in hyrax middens from southwestern Africa. Geology.

[26] Lim S., Chase B.M., Chevalier M., Reimer P.J. (2016). 50,000 years of vegetation and climate change in the southern Namib Desert, Pella, South Africa. Palaeogeogr. Palaeoclimatol. Palaeoecol..

[27] Burke A. (2013). Succulent plants on arid inselbergs. Flora.

[28] Hernández-Teixidor D., Santos I., Suárez D., Oromí P. (2020). The importance of threatened host plants for arthropod diversity: The fauna associated with dendroid *Euphorbia* plants endemic to the Canary and Madeira archipelagos. J. Insect Conserv..

[29] Taha A., Ettaqy A., El Mderssa M., Belaqziz M., Fokar M., Boukcim H., Zine El Abidine A., Abbas Y. (2023). Comprehensive review of morphological adaptations and onservation strategies of cactiform succulents: A case study of *Euphorbia* species in arid ecosystems. Biosyst. Divers..

[30] Al-Namazi A.A., Al-Khulaidi A.W.A., Algarni S., Al-Sagheer N.A. (2021). Natural plant species inventory of hotspot areas in Arabian Peninsula: Southwest Al-Baha region, Saudi Arabia. Saudi J. Biol. Sci..

[31] Grace O.M. (2019). Succulent plant diversity as natural capital. Plants People Planet.

